# *Mycobacterium tuberculosis* DosR is Required for Activity of the P_mbtB_ and P_mbtI_ Promoters under Hypoxia

**DOI:** 10.1371/journal.pone.0107283

**Published:** 2014-09-11

**Authors:** Lise J. Schreuder, Tanya Parish

**Affiliations:** 1 Queen Mary University of London, Barts and The London School of Medicine and Dentistry, London, United Kingdom; 2 TB Discovery Research, Infectious Disease Research Institute, Seattle, Washington, United States of America; Fundació Institut d'Investigació en Ciències de la Salut Germans Trias i Pujol. Universitat Autònoma de Barcelona. CIBERES, Spain

## Abstract

*Mycobacterium tuberculosis* has the ability to survive for extended periods of time under conditions of low oxygen, low pH, low iron and low nutrients. The mycobactins (*M. tuberculosis* siderophores) play a key role in scavenging iron from the environment and are induced in response to low iron in an IdeR-regulated manner. We demonstrate that the promoters of two mycobactin gene (*mbt*) operons are also expressed during adaptation to low oxygen, and that this expression is dependent on the DosR regulator. Up-regulation of *mbt* operons induced by low iron was not DosR-dependent. DosR is a member of a two component regulatory system which responds to oxygen availability. Deletion of the DosR regulator led to increased expression of bacterioferritin and increased capacity to grow under iron depletion. These data provide a link between the mycobacterial response to two conditions likely to be encountered *in vivo*, low iron and low oxygen.

## Introduction

*Mycobacterium tuberculosis* is the causative agent of tuberculosis (TB), a disease which kills over one million people a year [1]. A major concern is the ability of *M. tuberculosis* to persist as a latent infection without causing symptoms for decades, facilitating dissemination to distant locations and new hosts [2]. Understanding the mechanisms *M. tuberculosis* utilises to persist within the host are critical for determining new strategies to combat this highly successful pathogen.

Granulomas are the hallmark of TB, and are formed by infected and activated macrophages and other key host immune components, which results in isolating infected human cells and minimizing bacterial replication [3]–[6]. Within this highly organised environment bacterial growth is limited through deprivation of oxygen and nutrients, acidification and production of host factors such as nitric oxide [7], [8]. *In vitro* models of hypoxia have demonstrated that bacterial replication decreases and the bacilli enter a relatively quiescent and antibiotic-tolerant state where they can remain viable for years [9], [10]. The DosR-DosS/DosT two component regulatory system plays a pivotal role in mediating the adaptive response to hypoxia [11]–[15], regulating numerous genes [14], [16]–[18].

The uptake of iron is tightly controlled in prokaryotes, as failure to regulate intracellular iron would be lethal due to the ability of ferric iron to catalyse the production of oxygen radicals [19]. Iron is an essential element for growth and *M. tuberculosis* produces high-affinity iron siderophores (mycobactins) to sequester iron from the environment [20], [21]. Mycobactins are produced in response to iron limitation [22]; the mycobactin synthetic operon comprises 10 genes (*mbtA* to *mbtJ*), whose expression is up-regulated and induced in response to low iron in *M. tuberculosis*, as well as in activated macrophages [23]. Mycobactins are essential for virulence and survival, since disruption of their synthesis leads to impaired growth in macrophages or low iron medium [24], [25].

In *M. tuberculosis*, the key regulator of iron-dependent genes is IdeR, a member of the diphtheria toxin repressor (DtxR) family. IdeR is essential for *M. tuberculosis* survival in macrophages [22]. IdeR controls both the uptake of iron, by up-regulating *mbt* expression, and the intracellular storage of iron, by controlling expression of the bacterioferrritin BfrA [21], [26], [27].

The mycobactin synthetic gene cluster is comprised of ten genes *mbtA-J* putatively arranged as three transcriptional units. *MbtB-H* are divergently transcribed from *mtbAJ*, whereas *mbtI* is independently transcribed convergently with *mbtAJ*. The promoters upstream of *mbtB* and *mbtI* have been mapped by primer extension and functional IdeR boxes (to which IdeR binds) are located within each of these promoters [21]. No IdeR boxes or promoter motifs are found within the proposed operons, suggesting strongly that these are true transcriptional units.

We previously demonstrated that P_mbtB_ was active during hypoxic culture in *M. tuberculosis* [28], with high activity during adaptation to hypoxia (non-replicating phase stage 1; NRP1), but with activity decreasing over time as cells entered into non-replicating phase stage 2 (NRP2) [29]. Since MbtI is also involved in mycobactin synthesis, we wanted to see if it was under the same regulatory control as the *mbtB* operon. Our previous work had suggested that P_mbtI_ was induced two-fold by addition of tryptophan to the medium [30], but we had no data for hypoxia.

We were interested in the regulation of *mbtB* and *mbtI* promoters in *M. tuberculosis* in response to limited iron and to hypoxia. In this study we confirmed that up-regulation of activity is seen in response to iron limitation, and demonstrated that both promoters are active during adaptation to hypoxia. Surprisingly, the activity of both promoters was dependent on DosR under hypoxic conditions, but not under iron-limiting conditions, suggesting that dual regulation occurs. A DosR deletion mutant showed an increased ability to replicate under iron-limiting conditions and higher expression levels of bacterioferritin, suggesting that the intracellular iron storage levels are increased. These data suggest a link between two conditions, low oxygen and low iron, that are present *in vivo*.

## Materials and Methods

### Bacterial strains and culture conditions

*M. tuberculosis* H37Rv (ATCC 25618) was grown in Middlebrook 7H9 medium plus 10% AD (5% w/v bovine serum albumin, 2% w/v glucose) and 0.05% w/v Tween 80, (7H9-AD-Tw) or on Middlebrook 7H10 agar (Becton Dickinson) plus 10% v/v OADC (oleic acid, albumin, dextrose, catalase) supplement (Becton Dickinson). Aerobic growth was measured in 3 mL 7H9-AD-Tw in 16×125 mm glass cultures tubes with 8 mm magnetic stirrer bars stirring at 150 rpm. Low iron medium (MMT) was prepared containing 6 g L^−1^ Na_2_HPO_4_, 3 g L^−1^ KH_2_PO_4_, 0.5 g L^−1^ NaCl, 1 g L^−1^ NH_4_Cl and 0.0147 g L^−1^ CaCl_2_ supplemented with 0.05% w/v Tween 80 and 2% v/v glycerol and treated overnight with 5 g L^−1^ Chelex 100 (Sigma-Aldrich), 2 mM MgSO_4_ was added and the medium was filter-sterilised [21]. Medium for the Wayne model (DTA) was Dubos Broth Base (Becton Dickinson) supplemented with 10% v/v Dubos Medium Albumin (Becton Dickinson). Streptomycin was used at 40 µg mL^−1^ as required. The DosRΔ and DosRΔ complemented strains are described in [13].

### Promoter activity assays

The *M. tuberculosis* P_mbtB_-lacZ construct contained the 0.2 kbp upstream region of *mbtB* gene in the pSM128 vector upstream of the *lacZ* reporter [28], [31]. The P_mbtI_-lacZ construct contained 0.8 kbp upstream of *mbtI* (*trpE2*) gene in pSM128 [30]. *M. tuberculosis* was electroporated with 1 µg plasmid DNA [32] and transformants selected on plates containing streptomycin. Cell-free extracts and β-galactosidase assays were carried out as described [33].

### Iron depletion

*M. tuberculosis* cultures were grown to an OD_580_ of ∼1.0 in 100 mL MMT. To achieve iron starvation bacteria were harvested, washed in MMT, and sub-cultured into 100 mL MMT to an OD_580_ of 0.05 (10^7^ CFU/ml). Cells were grown to late log phase (OD_580_ 0.4–1.0) and sub-cultured to an OD_580_ of 0.05 for a minimum of two passages [21].

### Hypoxic cultures

*M. tuberculosis* strains were grown in oxygen-depleting conditions using the Wayne model of hypoxia [34]. Briefly, *M. tuberculosis* was grown in DTA to an OD_580_ of 0.4 and used to inoculate 17 mL of DTA in 20×125 mm glass culture tubes containing 8 mm magnetic stirrer bars to a theoretical OD_580_ of 0.004. Cultures were incubated at 37°C with stirring at 120 rpm. Methylene blue (0.1% w/v) was added to one culture tube per strain as an indicator of oxygen depletion.

### Quantitative RT-PCR

RNA was extracted from *M. tuberculosis* [35] WT, DosRΔ and DosR C′, and cDNA synthesised using random primers. Expression levels of *bfrA* and *bfrB* were determined using a TaqMan quantitative PCR assay. 7500 System SDS Software was used for the data analysis. The following primer/probe combinations were used. *BfrA*: primer pair bfrAF 5′ GTT GCT GGA TGG TTT GCC GAA CT 3′ and bfrAR 5′ TCT GGC GAT CGA ATA CGA CGT GTT and probe A 5′ TCG GCC AGA CGC TCC GCG A. *BfrB*: primer pair bfrBF 5′ TGT CGA AAT TCC CGG CGT AGA CA 3′ and bfrBR 5′ AGG AAC GCA CAG TCA CCG ACC A 3′ and probe B 5′ CCC GCG AGG CAC TGG CGC T. *SigA*: primer pair sigAF 5′ AAG CGG GCA GCC AAG AG 3′ and sigAR 5′ TCG AGT CGT CGG TCA CCT CAA 3′ and probe S 5′ TTG GCG GCC CGC TTG GCC. T.

Standard curves using genomic DNA were generated and used to calculate copy numbers. Relative gene expression for *bfrA* and *bfrB* was normalised to *sigA* copy number and assayed in biological triplicate for each strain.

## Results and Discussion

### P_mbtB_ and P_mbtI_ are expressed during adaptation to hypoxia

We determined if P_mbtI_ activity was dependent on oxygen status using the Wayne model of hypoxia. We compared promoter activity from both P_mbtB_ and P_mbtI_ using the same LacZ reporter gene to allow for a direct comparison of promoter strength. Our previous work suggested that LacZ was not a sensitive reporter for the early stages of hypoxia when cell numbers were limited, so we focused on measuring promoter activity after 7 days, after which time sufficient cells were present for the assay. We assayed activity over 42 days (14 days longer than previously assessed for P_mbtI_) [28].

**Figure 1:**
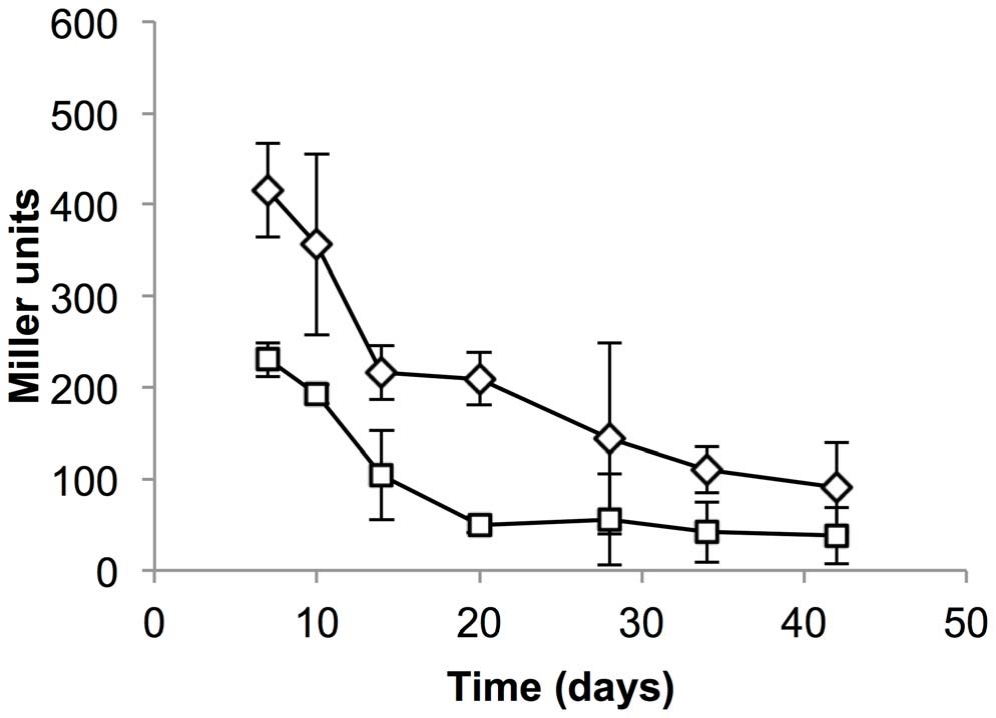
*mbtB* and *mbtI* promoters are induced in response to oxygen depletion in *M. tuberculosis*. P_mbtB_ and P_mbtI_ activity was measured as β-galactosidase activity from a LacZ reporter in cell-free extracts. Bacteria were cultured in the Wayne model of hypoxia. Diamond – *mbtB*; square – *mbtI*. Data are the mean and standard deviation from three independent transformations.

Both promoters were active during NRP1 (∼7 days), with decreasing activity over time as strains entered NRP2 (∼14 days) (Figure 1). As previously noted P_mbtB_ was highly active during NRP1 (>400 Miller units) (MU), which was reduced approximately four-fold as cells entered NRP2, although the promoter was still active during this latter phase. P_mbtI_ had a similar pattern of activity although the level of expression was about two-fold lower than P_mbtB_. The basal level of expression in late log phase in DTA was 259±7 for P_mbtB_ and 210±3 for P_mbtI_ (2 independent transformants), confirming that expression during adapatation was maintained and even transiently induced for P_mbtB_. These data suggest a transient requirement for mycobactin synthesis during the adaptation phase with down-regulation to a basal level not being complete until 28 days. No appreciable loss of cell viability was seen during the first 28 days (as measure by CFU – data not shown).

### P_mbtB_ and P_mbtI_ activity during hypoxia are DosR-dependent

**Figure 2:**
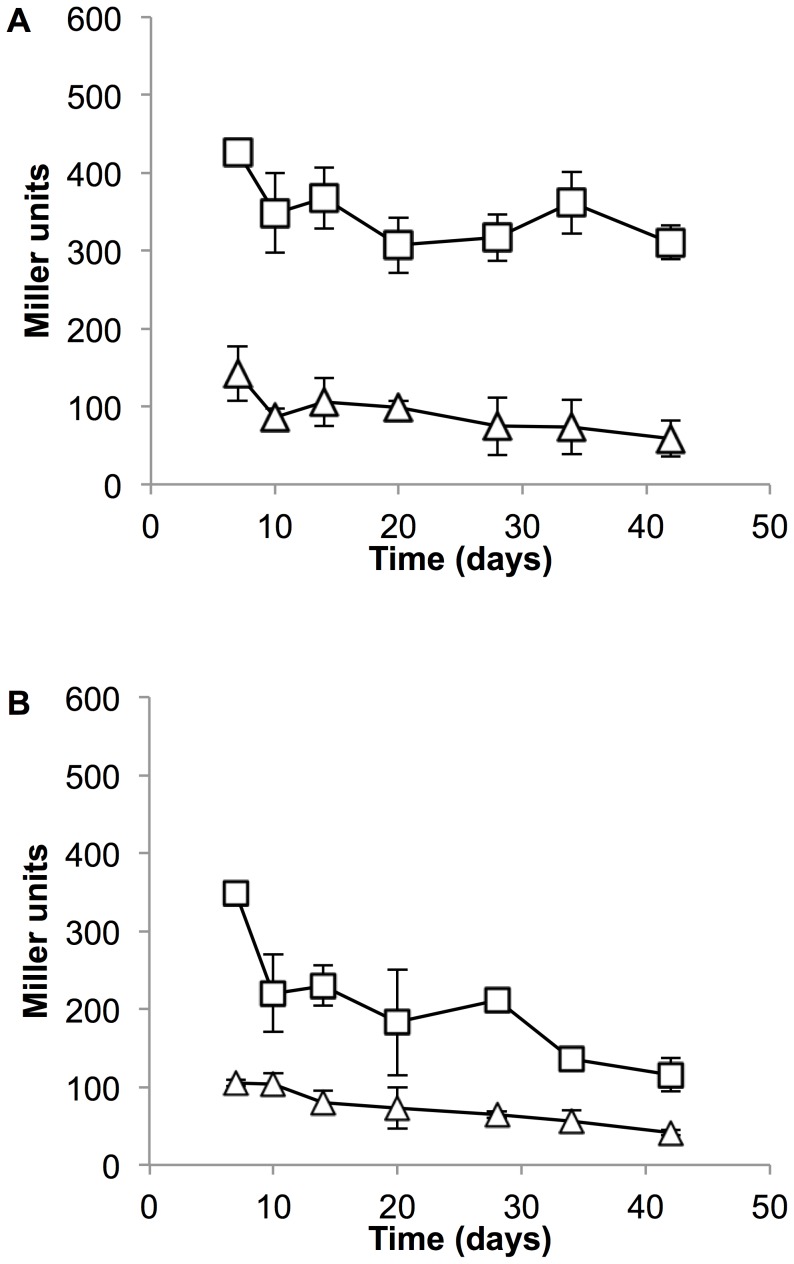
P_mbtB_ and P_mbtI_ activity during hypoxia is DosR-dependent. (A) P_mbtB_ and (B) P_mbtI_ activity was determined in bacteria during oxygen depletion in the Wayne model of hypoxia. Triangle – DosRΔ strain; square – DosRΔ complemented strain (C′). Data are the mean and standard deviation from three independent transformations.

The DosRST regulatory system controls the expression of many genes in response to hypoxia, and in particular the expression of genes during adaptation to hypoxia [7]. Since both mycobactin promoters were highly active during NRP1, we determined whether their activity was DosR-dependent. We used a deletion strain of DosR previously constructed, as well as a complemented strain – the deletion was an in-frame, unmarked deletion in DosR [13]. Interestingly expression of both promoters was reduced in the DosRΔ strain; both promoter retained activity, but were expressed at a basal level (100 MU) (Figure 2). Complementation restored activity and the expression pattern to wild-type levels confirming that promoter activity is DosR-dependent (Figure 2).

### Up-regulation of P_mbtB_ and P_mbtI_ in response to iron limitation is not DosR-dependent

**Figure 3:**
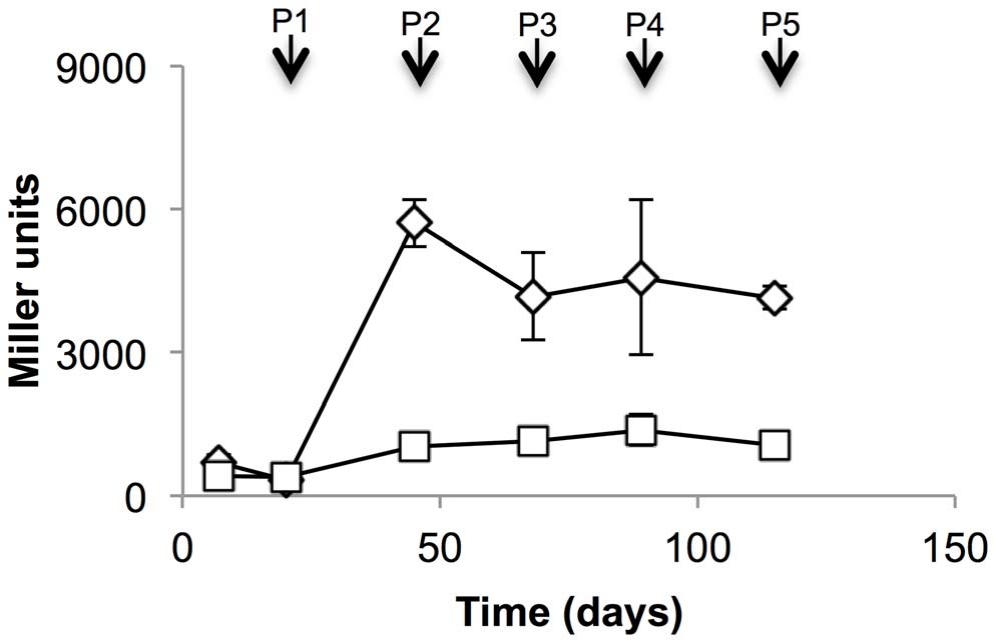
P_mbtB_ and P_mbtI_ are strongly induced in response to iron starvation in *M. tuberculosis*. (A) *mbtB* and (B) *mbtI* promoter activity was measured using a LacZ reporter in bacteria passaged in iron-depleted medium. β-galactosidase activity was measured in cell-free extracts. Diamond – *mbtB*; square – *mbtI*; P –passage number. Data are the mean and standard deviation from three independent transformations.

Since we had seen unusual regulation in response to hypoxia, we wanted to confirm that our promoter constructs showed iron-dependent expression. We measured promoter activity during iron depletion over five passages (115 days) in iron-free medium (Figure 3). Promoter activity was measured before each passage when cells were harvested for extracts and sub-cultured at the same time. Both promoters were induced in response to iron limitation after two passages (Figure 3) and activity remained at the induced level during subsequent passaging in iron-free medium. Activity in aerobic culture (7H9 medium) was 218±10 for P_mbtB_ and 402±15 for P_mbtI_ (two independent transformants); by passage 1, promoter activity was already being induced for P_s_ (708 Miller units) with maximal induction of 26-fold for P_mbtB_ and 3.3-fold for P_mbtI_. Once again, P_mbtB_ had much higher activity than P_mbtI_ although they showed similar trends of induction. In comparison to hypoxia, the induced levels were much higher - 6000 MU for P_mbtB_ and 1550 MU for P_mbtI_ as compared to the maximal levels under hypoxia of 415 and 230 MU respectively (in DTA medium).

**Figure 4:**
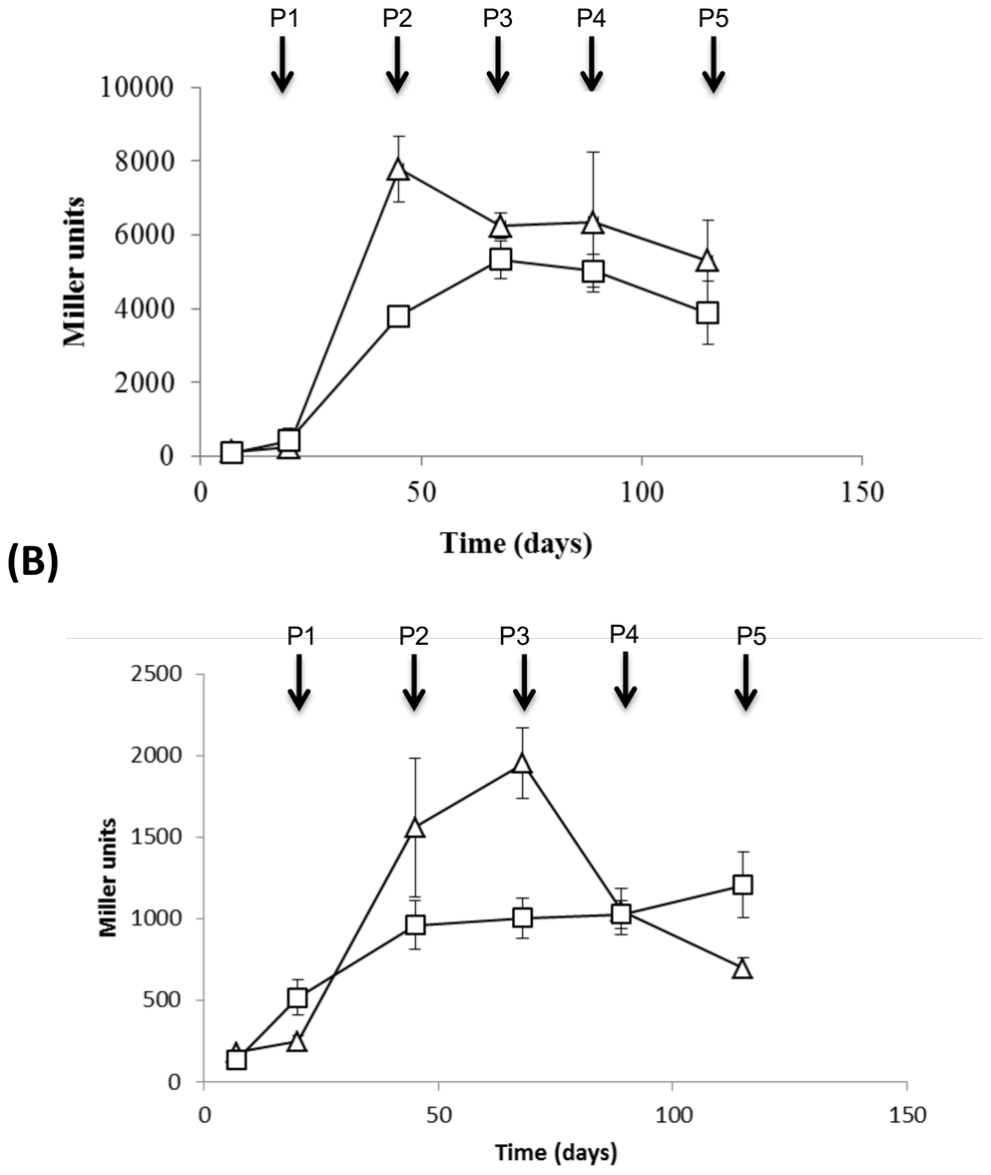
Induction of P_mbtB_ and P_mbtI_ in response to iron starvation is not DosR-dependent. Activity of (A) *mbtB* and (B) *mbtI* promoters was measured in *M. tuberculosis* passaged in iron-depleted medium. Triangle – DosRΔ strain; square - DosRΔcomplemented strain; P –passage number. Data are the mean and standard deviation from three independent transformations.

We determined whether any aspect of iron regulation for P_mbtI_ or P_mbtB_ was dependent on DosR. We measured promoter activity in the DosRΔ and DosR C′ strains under iron limitation (Figure 4). In both strains, significant increases in promoter activity were seen after the second passage (day 49) which were comparable to the levels seen in the wild-type. The maximal level of P_mbtB_ in the DosR C′ strain did not reach as high as the wild-type, but since the peak level is transient, we considered this was not a true difference and could result from a slightly earlier or later peak expression (since activity could only be assayed at the point of passage). For P_mbtI_, induction was seen in both strains after two passages to a similar extent and with the same pattern as the wild-type strain. These data confirmed that DosR was not required for iron-dependent expression of either promoter.

### DosR deletion leads to increased growth during iron depletion

Although DosR status did not affect iron-dependent expression of mycobactin genes, we were interested to see if it had any effect on growth during iron limitation. Earlier studies suggested differences in growth of *M. tuberculosis* mutants under different conditions, such as low iron and pH [36], [37].

**Figure 5:**
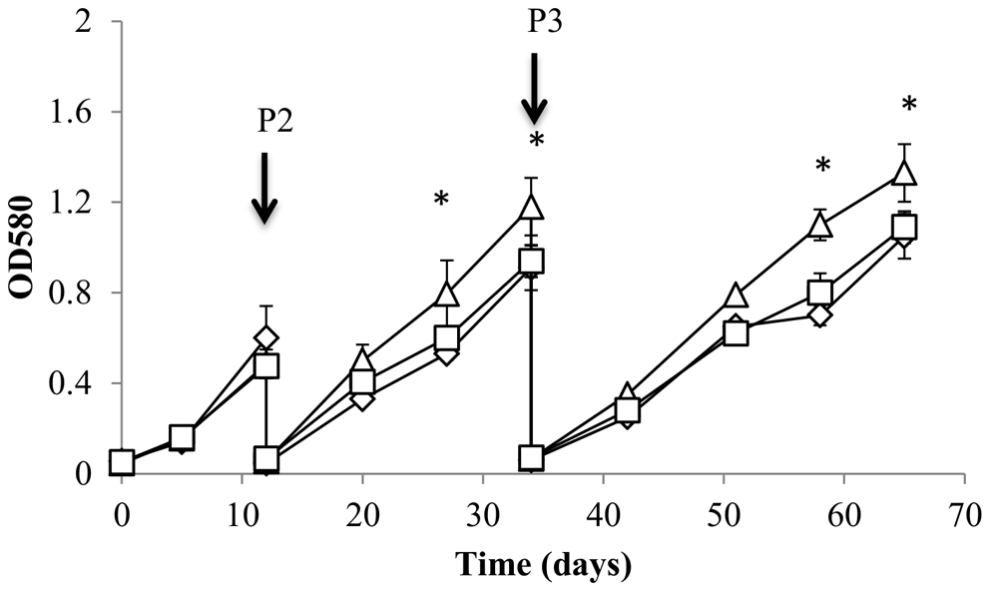
Growth of the DosRΔ strain is more robust during iron depletion. Bacterial growth was monitored over three passages (P1-3) in iron-depleted medium. Diamond – wild type strain; triangle – DosRΔ strain; square – DosRΔ complemented strain. Data are the mean and standard deviation from three independent cultures. * p<0.05 using Student's t-test comparing the DosRΔ and wild type strain.

We passaged the DosRΔ strain in low iron medium and measured growth rate over several passages (Figure 5). The wild-type and complemented strains showed a decrease in growth rate during passaging, taking longer to reach the same OD with each passage. This reduction in growth rate was less marked in the DosRΔ strain, which grew significantly faster than the wild-type after extended iron depletion. Cultures were grown under highly aerated conditions (in roller bottles) and passaged before reaching stationary phase; no difference was seen in the growth rate between early and late log phase, making it unlikely that oxygen limitation was a factor.

### Bacterioferritin expression is elevated in the DosRΔ strain

One possibility for this increased capacity to withstand iron depletion of the DosRΔ strain is that intracellular iron storage levels are higher, such that internal iron depletion takes longer (more cell divisions can occur before iron is limiting). *M. tuberculosis* has two bacterioferritins (BrfA and BrfB), which play an important role in iron storage and homeostasis [26]. We measured the expression levels of *bfrA* and *bfrB* in WT, DosRΔ and DosR C′ *M. tuberculosis* grown under iron-replete conditions (Table 1). We selected this condition as being representative of the initial inoculum for the iron depletion experiment, if cells had higher levels of iron at the beginning, they might be expected to sustain growth for longer. We saw similarly low expression levels of *bfrA* in all three strains. However, the level of *bfrB* expression differed, with the expression levels in DosRΔ being significantly higher than in the WT and complemented strains. This suggests a greater capacity for iron storage in the DosRΔ strain, as compared to the WT strain (Table 1).

**Table 1:** Expression of *bfrA* and *bfrB* in *M. tuberculosis* strains.

Strain	*bfrA*	*bfrB*
**WT**	2.1+/−0.5	6.2+/−1.6
**DosRΔ**	2.9+/−0.2	35.4+/−9.5
**C′ DosR**	3.0+/−0.03	18.7+/−3.9

RNA was isolated from *M. tuberculosis* strains grown under aerobic, iron-replate conditions. Levels of *bfrA* and *bfrB* expression were measured using quantitative PCR and normalised to to SigA expression. Data are the mean and standard deviation (SD) from three independent transformations.

## Conclusions

IdeR is the major regulator of iron-dependent gene expression and controls the expression of the *mbt* operons in response to iron availability. We have demonstrated a second level of regulation mediated by DosR in response to oxygen availability. It is not clear whether this is mediated directly by DosR as transcriptional regulator, although there are no “Dos” motifs located upstream of the *mbt* promoters, so it seems unlikely that DosR would regulate at this level. Analysis of the regulatory networks of *M. tuberculosis* using TB Database [38] reveals a possible connection between DosR and IdeR, but only at the fourth level. However, there is a direct link between DosR and Rv2034, a member of the IdeR regulon. Future work could help to elucidate the regulatory mechanism involved.

An alternative hypothesis is that the internal levels of iron differ between WT and DosRΔ *M. tuberculosis*. The higher level of activity of both promoters in the deletion strain suggests that there may be higher levels of mycobactin, and by extension more acquisition of iron from the environment. The high level of *bfrB* in DosRΔ, suggests a greater potential for storing iron and could explain the increased survival and growth in iron-depleted medium. This ability may also partly explain the increased virulence of DosRΔ [13], as the mutated strain might better survive *in vivo* with a larger iron reservoir.
